# Effect of Machine Entropy Production on the Optimal Performance of a Refrigerator

**DOI:** 10.3390/e22090913

**Published:** 2020-08-20

**Authors:** Michel Feidt, Monica Costea

**Affiliations:** 1 LEMTA, URA CNRS 7563, University of Lorraine, 2, avenue de la Forêt de Haye, 54518 Vandoeuvre-lès-Nancy, France; michel.feidt@univ-lorraine.fr; 2 Department of Engineering Thermodynamics, Engines, Thermal and Refrigerating Equipment, University Politehnica of Bucharest, 313 Splaiul Independentei, 060042 Bucharest, Romania

**Keywords:** refrigerator, optimization, minimum energy expense, heat transfer conductance allocation, production of entropy

## Abstract

The need for cooling is more and more important in current applications, as environmental constraints become more and more restrictive. Therefore, the optimization of reverse cycle machines is currently required. This optimization could be split in two parts, namely, (1) the design optimization, leading to an optimal dimensioning to fulfill the specific demand (static or nominal steady state optimization); and (2) the dynamic optimization, where the demand fluctuates, and the system must be continuously adapted. Thus, the variability of the system load (with or without storage) implies its careful control-command. The topic of this paper is concerned with part (1) and proposes a novel and more complete modeling of an irreversible Carnot refrigerator that involves the coupling between sink (source) and machine through a heat transfer constraint. Moreover, it induces the choice of a reference heat transfer entropy, which is the heat transfer entropy at the source of a Carnot irreversible refrigerator. The thermodynamic optimization of the refrigerator provides new results regarding the optimal allocation of heat transfer conductances and minimum energy consumption with associated coefficient of performance (COP) when various forms of entropy production owing to internal irreversibility are considered. The reported results and their consequences represent a new fundamental step forward regarding the performance upper bound of Carnot irreversible refrigerator.

## 1. Introduction

The refrigeration machine continues to play a vital role in the field of engineering applications, proven by numerous papers published in international journals, some of them specifically dedicated to this topic.

By examining recently published scientific works regarding reverse cycle machines, one remarks that more papers are devoted to refrigerating machines than to heat pumps [1,2,3,4,5,6,7,8,9,10], which also corresponds to current applications that are more and more developed; that is, refrigeration freezing and deep-freezing in the food industry, air conditioning, heat pumps, or medical applications.

To meet the consumer requirements regarding the performance and energy expense, the optimization of reverse cycle machines appears as an important task. The optimization procedure could follow two trends depending on the machine operation mode. Thus, the first one is relative to the nominal operation conditions and provides optimal dimensioning of the machine subsystems (design optimization). The second one is associated with control-command of the reverse cycle systems owing to the variability of ambient and operation conditions (dynamic optimization).

Many studies in the literature are concerned with specific improvement of reverse cycle machines, most recently dealing with topical applications, namely, thermoelectric cooler [11], vortex tube design models [12], electronic cooling with nanofluids [13], ground source heat pumps applications [14], electrocaloric refrigeration [15], or magnetic refrigeration [16].

Our approach focuses on a global optimization from a thermodynamic point of view, even if the thermo-economic optimizations [17,18,19,20,21,22,23,24] are also important and indispensable from an engineering point of view.

Note that, in terms of the thermodynamic approach, finite time thermodynamics (FTT) optimization and entropy generation minimization [4,18] reported important results in the recent past. Nevertheless, these approaches remained limited to endo-reversible thermodynamics tools [4,25,26].

Some insight aiming to get closer to real operation of reverse cycle machines has been developed using linear irreversible thermodynamics [27].

The purpose of this paper is to complete the previous reviews [1,2]. Thus, an extension of a new modified modelling of Carnot irreversible refrigerator recently developed [4] is proposed, aiming to provide optimal physical dimensions of the refrigerator, mainly optimal allocation of heat transfer characteristics between source (sink) and machine (internal fluid cycled). The model developed in [4] has considered the coupling between thermostats and machine by an energy balance between source (sink) and cycled medium, but limited only to the endo-reversible case. This methodology has introduced a supplementary physical quantity, namely the heat transfer entropy Δ*S* with reference to source for the refrigerator. In the endo-reversible case [4], the answer is analytical. Furthermore, these results were extended to a refrigerator with internal irreversibility characterized by production of entropy Δ*S_I_*, which was supposed as a *constant parameter* [5]. The proposed modelling in [5] explored the performance of Chambadal [28] and Curzon-Ahlborn [29] cycles transposed from engine in refrigerators by gradually emphasizing the influence of irreversibilities through internal or total entropy productions, and through *irreversibility degrees* introduced by Novikov [30] and *irreversibility ratios* introduced by Ibrahim et al. [31]. The results have shown that Chambadal’s model optimum coincides with the equilibrium thermodynamics one, namely, the non-existence of minimum energy consumption, while the optimization of the Curzon-Ahlborn model relates the external temperatures to the finite dimensions of the machine (*G_H_*, *G_C_*) that have to be optimally allocated. Analytical expressions of the optimal distribution of the finite heat transfer conductances *G_H_* and *G_C_* leading to the minimum energy expense were derived for Δ*S* and Δ*S_I_ constant parameters* and the total finite dimensions *G_T_* fixed.

The present paper presents the thermodynamics optimization of the Carnot endo-irreversible refrigerator, providing new results regarding the optimal allocation of heat transfer conductances and minimum energy consumption and associated coefficient of performance (*COP*) when various forms of entropy production owing to internal irreversibility are considered. It uses the same methodology, but further developed for the case when the minimization of *mean power consumption* is sought (Section 2.2). The new concept of *production entropy action* is introduced, being analogous to *energy action* used in classical mechanical.

A step further consists of a quasi-Chambadal refrigerator modeling analysis. In this case, the source is considered finite, determining the *heat transfer across a temperature gap*, while the sink (atmosphere) remains in perfect thermal contact with the cycled fluid. The modeling of the heat transfer at the cold source in the quasi-Chambadal refrigerator is developed for two different cases, once considering the heat transfer coupling constraint, and then without coupling. The effect of *various forms of functional dependence of the entropy production* on the results reveals new expressions of *COP* and mean work per cycle.

The Curzon-Ahlborn refrigerator model extends the study achievements. It is more complete and allows modeling without heat transfer coupling at source and sink (classical appraisal), or with coupling. Regarding the energy optimization results, the last case leads to a new optimal allocation of heat transfer conductance, with corresponding *new upper bounds* for the *COP* and minimum work expense in endo-reversible cycle, *min W_endo-rev_* (Section 3.2).

Numerical results are illustrated when the production of entropy depends on temperatures and reference heat transfer entropy. An asymptotic solution is proposed in the low irreversibility case.

## 2. Thermodynamic Cycles of Two Thermal Reservoirs Machines

### 2.1. The Standard Reverse Cycles

**Figure 1:**
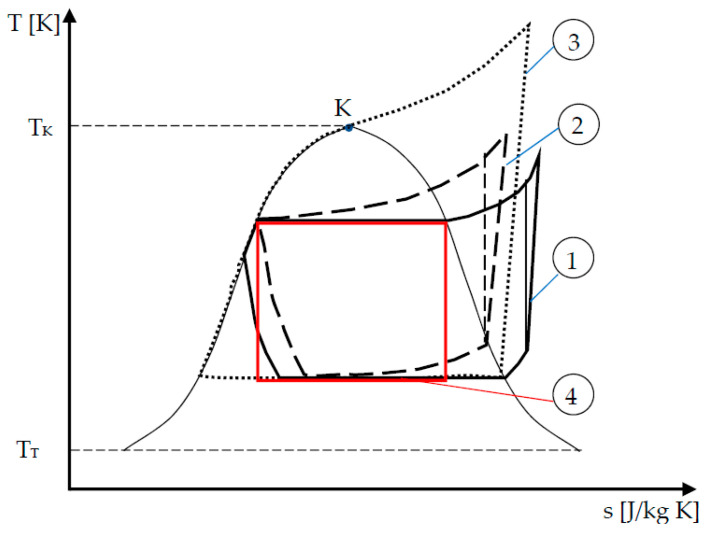
Cycles of vapor compression machines illustrated in *T–s* diagram.

The topic developed here is only concerned with the reverse cycle machines coupled with one heat source and a cold sink. Generally, these machines are vapor compression machines according to cycle 1 illustrated in Figure 1. Owing to technological constraint, the cycle is composed of a compression of superheated vapor that is followed by desuperheating of vapor. Then, a condensation of the cycled fluid occurs as well as, generally, a subcooling of the saturated liquid before entering the expansion valve, where an isenthalpic transformation occurs. The last transformation closing the cycle consists of fluid vaporization and superheating of vapor. Note that this kind of cycle is efficient only in a specific interval characteristic of a pure fluid, referring here to the temperature variation range [*T_T_*, *T_K_*], with *T_T_* (*T_K_*) triple point (critical point) temperature.

If the fluid is a mixture, the corresponding cycle is subject to a glide of temperature in the liquid vapor domain, thus the corresponding cycle is near to a Lorenz cycle (cycle 2 in Figure 1).

If a more important variation of temperature is needed, supercritical (or transcritical) cycles are possible (cycle 3). In this configuration, the variation of temperature at the hot side could be huge.

In any case, the problem is to develop the more efficient reverse cycle machines satisfying specific constraints or not. In the paper, only the limiting case of Carnot configuration (cycle 4) aiming at determining performance upper bound of the two reservoirs machines will be considered.

Classically, the equilibrium thermodynamics is concerned with determining the upper bound efficiency of a cycle. For a reverse cycle machine, this efficiency is characterised by the coefficient of performance (*COP*) relative to the refrigerator *COP_REF_* or to the heat pump *COP_HP_*.

If the cold source is a thermostat at temperature *T_CS_*, and the hot sink is a thermostat at temperature *T_HS_*, one gets the well-known result depending only on these two temperatures:

(1) $$COP_{REF\;rev}=\frac{T_{CS}}{T_{HS}-T_{CS}}=\frac{T_{CS}}{\Delta T_{S}},$$

(2) $$COP_{HP\;rev}=\frac{T_{HS}}{T_{HS}-T_{CS}}=\frac{T_{HS}}{\Delta T_{S}},$$

with Δ*T_S_* = *T_HS_* − *T_CS_*.

These formulas determine the upper bound of *COP*s under reversible operation conditions of the system consisting of source–machine–sink. Nevertheless, these quasi static conditions are associated with infinite time duration of the processes, thus the corresponding heat transfer rate at the cold or hot side of the machine is null. This is the reason for which the modelling hereafter will consider the endo-irreversible cycle condition focused on refrigeration to illustrate the methodology and the obtained results. The same modelling could be adapted for heat pump configuration. It is under development by our research group and is in due course of publication.

### 2.2. Endo-Irreversible Carnot Refrigerator

**Figure 2:**
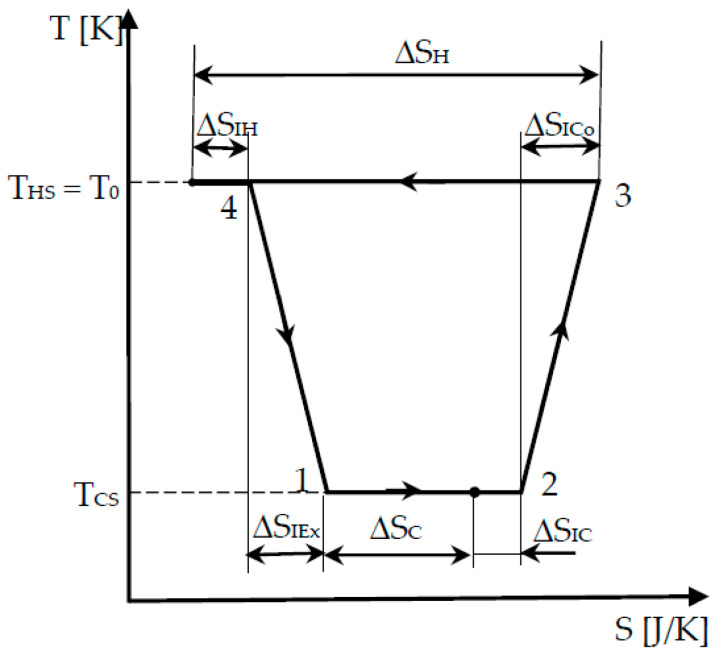
Carnot endo-irreversible refrigerator.

The endo-irreversible Carnot machine configuration is represented in Figure 2 and can also be named the exo-reversible Carnot refrigerator [5], as there is no heat transfer at finite temperature gap at the cold and hot side of the machine.

The irreversible cycle is identified from state 1 (beginning of the low temperature isotherm), 2 (exit of the low temperature isotherm and beginning of the adiabatic compression), 3 (end of the *irreversible* adiabatic compression and beginning of the high temperature isotherm), and 4 (exit of the high temperature isotherm and beginning of the *irreversible* adiabatic expansion, up to state 1).

Figure 2 also shows the internal irreversibility of the cycle by the corresponding production of entropy on the two isothermal transformations Δ*S_IC_*, Δ*S_IH_* and on the adiabatic compression and expansion, Δ*S_ICo_*, Δ*S_IEx_*, respectively. The sum of these four terms is the cycle production of entropy, Δ*S_I_*.

In this configuration, one supposes that thermal loss between hot and cold side does not exist. This is a favorable case allowing to estimate the upper bound for the *COP* corresponding to the minimum of work expense.

The Carnot endo-irreversible model is presented for a cycle with the constraint relative to temperatures that imposes $T_{HS}=T_{0}>T_{CS}$ (*T*_0_, ambient temperature).

The energy balance over a cycle is expressed as follows:

(3) $$\left|W\right|=\left|Q_{H}\right|-Q_{C},$$

where *W* is the work input in the cycle and $Q_{H},\;\left(Q_{C}\right)$ are the heat delivered at the hot sink (heat received at the cold source) by the working fluid. They are expressed as follows:

(4) $$\left|Q_{H}\right|=T_{HS}\left|\Delta S_{H}\right|=T_{0}\left|\Delta S_{H}\right|,$$

(5) $$Q_{C}=T_{CS}\Delta S_{C},$$

The entropy balance is expressed as follows:

(6) $$\left|\Delta S_{H}\right|=\Delta S_{C}+\Delta S_{I},$$

where $\Delta S_{H}$, heat transfer entropy released at the hot side during the cycle;

$\Delta S_{C}$, heat transfer entropy received at the cold side by the cycle medium;

$\Delta S_{I}$, the total entropy production during the cycle. It is the sum of four entropy productions corresponding to each transformation of the cycle (see Figure 2), successively indicated by $\Delta S_{IC},\Delta S_{ICo},\Delta S_{IH},\Delta S_{IEx}$ such that

(7) $$\Delta S_{I}=\Delta S_{IC}+\Delta S_{ICo}+\Delta S_{IH}+\Delta S_{IEx}.$$

By combining Equations (3)–(6) and using as reference the heat transfer entropy chosen at the useful cold side ($\Delta S_{C}$) that will be simply denoted by ΔS, one gets the following:

(8) $$\left|W\right|=\Delta S(T_{HS}-T_{CS})+T_{0}\Delta S_{I}.$$

This relation corresponds to the Gouy–Stodola theorem applied to the refrigerator, stating that the minimum of energy expense corresponds to the minimum of entropy production. In fact, this condition is achieved for the reversible or quasi static operation.

Equations (1), (5), and (8) provide the expression of the corresponding *COP* as follows:

(9) $$\frac{1}{COP}=\frac{\left|W\right|}{Q_{C}\;}=\frac{1}{COP_{rev}}+\frac{T_{0}}{T_{CS}}\cdot \frac{\Delta S_{I}}{\Delta S}.$$

It results from Equation (9) that the real *COP* is a decreasing function of two ratios:

- the *intensive* ratio $\frac{T_{0}}{T_{CS}}$;
- the *extensive* ratio $\frac{\Delta S_{I}}{\Delta S}$ (corresponding to the one introduced for Carnot engine by Novikov [21]).

Considering the power expense of the refrigerator, one can express its mean value over the cycles as follows:

(10) $$\bar{\dot{W}}=\frac{\left|W_{rev}\right|+T_{0}\Delta S_{I}}{\tau },$$

where τ, time duration of the cycle.

The quasi static case is well approximated by an entropy production inversely proportional to τ:

(11) $$\Delta S_{I}=\frac{C_{I}}{\tau }.$$

Note that Equation (11) provides the important definition of *C_I_*, which represents a *production entropy action* with the unit (J·s/K). It is analogous to *energy action* used in classical mechanical.

By combining Equations (10) and (11), the mean power appears as a decreasing function of τ. Contrarily to the engine performance, an optimum of power related to the finite *W_rev_* imposed does not exist.

Nevertheless, when the mean power ${\bar{\dot{W}}}_{rev}$ is provided for the refrigerating machine, the energy expense becomes the following:

(12) $$\left|W\right|={\bar{\dot{W}}}_{rev}\tau +\frac{C_{I}T_{0}}{\tau }.$$

In this case, by considering the second-order equation of (12), a minimum of mechanical energy expense is obtained for τ*:

(13) $$\tau^{*}=\sqrt{\frac{C_{I}T_{0}}{{\bar{\dot{W}}}_{rev}}},$$

and it depends on the square root of the production entropy action:

(14) $$\min\left|W\right|=\tau \sqrt{{\bar{\dot{W}}}_{rev}C_{I}T_{0}}.$$

A paradox appears here, because when $C_{I}\to 0$, $\tau^{*}\to 0$ and minW→0.

### 2.3. Quasi-Chambadal Refrigerator

**Figure 3:**
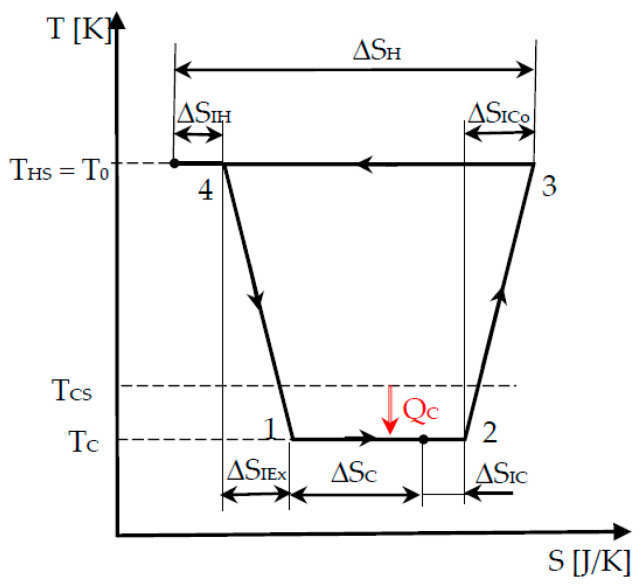
Cycle representation of Chambadal’s refrigerator.

The corresponding cycle focuses on the *useful heat transfer at the cold source* and supposes perfect heat transfer at the hot sink, which is the environment at temperature *T*_0_ (Figure 3).

Thus, the major advance in the modelling compared with the previous case (Section 2.2) is given by the heat transfer *Q_C_*, which is performed at *a temperature gap* (*T_CS_−T_C_*) in Chambadal refrigerator cycle. This leads to two potential approaches, so-called without coupling and with coupling at the cold source. The difference between them is provided by the way the heat transfer *Q_C_* is expressed, once using the heat transfer entropy, and then by involving the heat transfer conductance relative to the heat exchanger at the refrigerator cold side.

#### 2.3.1. Modelling without Coupling at the Cold Source

The modelling is analogous to the previous one (Section 2.2).

The same Δ*S_C_* remains as reference heat transfer entropy, but the cold side temperature at which heat is transferred to the cycled medium is *T_C_* instead of *T_CS_*. Thus, the heat transfer to the cold side is expressed as follows:

(15) $$Q_{C}=T_{C}\Delta S.$$

The mechanical energy needed becomes the following:

(16) $$\left|W\right|=\left(T_{0}-T_{C}\right)\Delta S+T_{0}\Delta S_{I}.$$

Note that, at this time, *W* depends on temperature *T_C_* and not *T_CS_*. As *T_C_* < *T_CS_*, it turns out that the necessary heat transfer involves a higher mechanical expense. In Equation (16), *W* appears as a decreasing function of *T_C_*, if $\Delta S_{I}$ is a considered parameter. The results corresponding to this assumption were reported in [5]. However, $\Delta S_{I}$ could also be a function of other properties (temperature, size).

From current knowledge, the production of entropy owing to internal irreversibility of the converter does not have a general analytical expression, as the heat transfer entropy has. Some attempts have been reported [32,33] in the finite speed thermodynamics approach of irreversible Stirling machines and Carnot-like refrigerating machines. Nevertheless, the derived analytical expression of the entropy production remains very much dependent on the study assumptions.

The purpose of the present modelling is to emphasize analytically the effect of internal irreversibility on refrigerator performance. Thus, the simplest forms of the functional dependence of $\Delta S_{I}$ were chosen to show the influence of *intensity* (Δ*T*) or *extensity* (Δ*S*) or the combination of the two. They are considered to hold mainly in the vicinity of the optimum found.

The most common forms for production of entropy used in this analysis are as follows:

(a) proportionality to the reference useful heat transfer entropy (*extensity*):
  (17) $$\Delta S_{I}=d_{I}\Delta S,$$
  with *d_I_* irreversibility degree. Thus, $\Delta S_{I}$ is a function of the reference extensity.
(b) proportionality to the gradient of available *intensity* $\left(T_{0}-T_{C}\right)$ for the converter:
  (18) $$\Delta S_{I}=s_{IL}\left(T_{0}-T_{C}\right).$$
(c) the third functional dependence is a combination of the two preceding ones (proportionality to the reference useful reversible energy):
  (19) $$\Delta S_{I}=k_{IL}\;\Delta S\;\left(T_{0}-T_{C}\right).$$

It is easy to show that, for these three general linear forms of the function regarding the entropy production of a refrigerator, this production is decreasing when $T_{C}$ increases or with the increase in $\left(T_{0}-T_{C}\right)$ as well as in ΔS, and obviously the increase in the three corresponding irreversibility parameters $\left(d_{I};s_{IL};k_{IL}\right)$ introduced by Equations (17)–(19). Thus, it is obvious that there is no *minW* in this case.

The general formulation of *COP* results from (15) and (16) as follows:

(20) $$\frac{1}{COP}=\frac{\left|W\right|}{Q_{C}}=\frac{T_{0}}{T_{C}}\left(1+\frac{\Delta S_{I}}{\Delta S}\right)-1,$$

which, compared with *COP* for the reversible cycle,

(21) $$\frac{1}{COP_{rev}}=\frac{T_{0}}{T_{CS}}-1,$$

emphasizes a correcting factor depending on temperatures, heat transfer entropy, and production of entropy:

(22) $$F\left(T_{CS},T_{C},\Delta S_{I},\Delta S\right)=\frac{T_{CS}}{T_{C}}\left(1+\frac{\Delta S_{I}}{\Delta S}\right),$$

which clearly shows the effect of irreversibility on the Chambadal refrigerator *COP*:

(23) $$\frac{1}{COP}=\frac{T_{0}}{T_{CS}}F-1=\left(\frac{1}{COP_{rev}}+1\right)F-1.$$

Obviously, the increase of *T_CS_* and $\Delta S_{I}$ will decrease the *COP*, while it will increase with *T_C_* and ΔS.

#### 2.3.2. Modelling with Coupling at the Cold Source (Coupled Chambadal Refrigerator)

The Chambadal’s model introduces a coupling, but only at the cold side of the system. For a linear heat transfer law, we define a new general physical quantity *G_C_* (J/K), a heat transfer conductance relative to the cold heat exchanger introduced by the following:

(24) $$Q_{C}=T_{C}\Delta S=G_{C}\left(T_{CS}-T_{C}\right).$$

The consequence of this new constraint is that $T_{C}$ changes from an independent variable to a determined one according to the following:

(25) $$T_{C}=\frac{G_{C}T_{CS}}{G_{C}+\Delta S}.$$

Using the same approach as in Section 2.3.1, one can show that the consumed mechanical energy W is increasing with ΔS as well as with $d_{I}\;or\;\left(s_{IL},k_{IL}\right)$, but is decreasing with $G_{C}$, whichever form of the entropy production function is considered.

The coupling at the cold source will modify the *COP* (Equation (23)) as well by the new correcting factor F expressed as follows:

(26) $$F=\frac{G_{C}+\Delta S_{C}}{G_{C}}\left(1+\frac{\Delta S_{I}}{\Delta S}\right).$$

In the endo-reversible case ($\Delta S_{I}=0)$, one gets the following:

(27) $$\frac{1}{COP_{endo-rev}}=\frac{T_{0}}{T_{CS}}\frac{G_{C}+\Delta S}{G_{C}}-1.$$

Equation (27) represents a new upper bound of *COP* of the endo-reversible cycle that results from the coupling at the cold side of Chambadal refrigerator in the modelling based on heat transfer entropy and production of entropy. It clearly shows that the ratio $\frac{\Delta S}{G_{C}}$ makes the difference from the well-known expression of *COP* for reversible cycle (Equation (21)) by decreasing the *COP* when it rises.

## 3. The Curzon-Ahlborn Refrigerator

**Figure 4:**
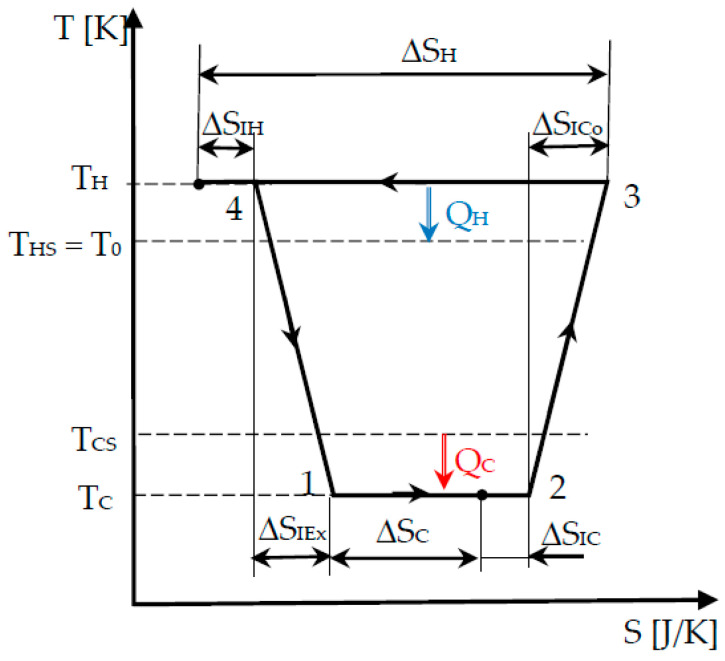
Cycle representation of Curzon-Ahlborn’s refrigerator.

This is the more comprehensive modelling that is examined here as it completes the Chambadal approach by considering the heat transfer across a temperature gap to the hot sink as well. Thus, the cycle illustrated in Figure 3 is modified in Figure 4 by considering the real heat transfer conditions, namely, different temperature of the source and cycle medium at the hot side, respectively.

### 3.1. Modelling without Coupling Constraints

The methodology is the same as the one applied in Section 2.3.1, but, owing to the formal symmetry between hot and cold side of the system (see Figure 4), it appears this time that the results depend on *T_H_* value. The entropy production laws (18) and (19) become the following:

(28) $$\Delta S_{I}=s_{IL}\;\;\left(T_{H}-T_{C}\right),$$

(29) $$\Delta S_{I}=k_{IL}\;\Delta S\;\left(T_{0}-T_{C}\right).$$

The energy expense is increasing with irreversibilities parameters $\left(d_{I},s_{IL},\;k_{IL}\right)$, and with Δ*S* and *T_H_*, but it is decreasing with *T_C_*.

### 3.2. Modelling with Coupling Constraints

The same coupling constraint (24) used in the Chambadal model (Section 2.3.2) is conserved here as well, to which the constraint owing to heat transfer between the hot side of the cycle and heat sink is added:

(30) $$\left|Q_{H}\right|=T_{H}\left|\Delta S_{H}\right|=G_{H}\left(T_{H}-T_{HS}\right).$$

Combining the entropy balance given by Equation (4) with Equation (30) allows to conserve the same heat transfer reference entropy, thus one gets the following:

(31) $$T_{H}=T_{HS}\frac{G_{H}}{G_{H}-\left(\Delta S+\Delta S_{I}\right)}.$$

Note that, if $\Delta S_{I}$ is no longer a constant parameter, as it is expressed by the functions (28) and (29), $T_{H}$ appears in Equation (31) as a function of $T_{HS}$, $G_{H}$, and ΔS, as well as of $T_{C}$, according to Equation (25). This corresponds to a strong coupling and will be developed in the following section.

Nevertheless, we want to detail and comment on the case where $\Delta S_{I}$ is considered a constant parameter, as well as the importance of the finite physical dimension constraint, owing to the heat transfer limitations of the system:

(32) $$G_{H}+G_{C}=G_{T},$$

where $G_{T}$ represent the total heat transfer conductance to be optimally allocated between the hot and cold side.

This completes the preceding works done on refrigerators [5], as well as on engines [34].

Variational calculus furnishes the following analytical results:

(33) $$G_{C}^{*}=\frac{\Delta S}{\left(\Delta S+\Delta S_{I}\right)\sqrt{T_{HS}}+\Delta S\sqrt{T_{CS}}}\left[\left(G_{T}-\Delta S-\Delta S_{I}\right)\sqrt{T_{CS}}-\left(\Delta S+\Delta S_{I}\right)\sqrt{T_{HS}}\;\right],$$

(34) $$G_{H}^{*}=\frac{\Delta S+\Delta S_{I}}{\left(\Delta S+\Delta S_{I}\right)\sqrt{T_{HS}}+\Delta S\sqrt{T_{CS}}}\left[\left(G_{T}+\Delta S\right)\sqrt{T_{HS}}+\Delta S\sqrt{T_{CS}}\;\right].$$

The corresponding min|W| is as follows:

(35) $$min\left|W\right|=\frac{1}{G_{T}-\Delta S_{I}}\left\{G_{T}\left[T_{HS}\left(\Delta S+\Delta S_{I}\right)-T_{CS}\Delta S\right]+\Delta S\left(\Delta S+\Delta S_{I}\right){\left(\sqrt{T_{HS}}+\sqrt{T_{CS}}\right)}^{2}\right\}.$$

This function that corresponds to the optimal distribution of *G_H_* and *G_C_* is increasing with Δ*S* and $\Delta S_{I}$, and decreasing with $G_{T}$. However, one notes that the increase of *G_T_* raises the cost of the system, leading to an economic compromise between capital expenditure (designing) and operational cost (energy consumption over the system life).

To complete the results, the combination of Equations (24), (30), (33), and (34) gives the *COP* corresponding to min|W| conditions as follows:

(36) $$\frac{1}{COP\;\left(min\left|W\right|\right)}=\sqrt{\frac{T_{HS}}{T_{CS}}}\cdot \frac{\Delta S+\Delta S_{I}}{\Delta S}\cdot \frac{G_{T}\sqrt{T_{HS}}+\Delta S\left(\sqrt{T_{HS}}+\sqrt{T_{CS}}\right)}{G_{T}\sqrt{T_{CS}}-\left(\Delta S+\Delta S_{I}\right)\left(\sqrt{T_{HS}}+\sqrt{T_{CS}}\right)}-1=\frac{T_{HS}}{T_{CS}}F-1,$$

where the correction factor expression is much more complex than those derived for Chambadal refrigerator (Equations (22) and (26)) by involving intensities and extensities as well:

(37) $$F=\sqrt{\frac{T_{CS}}{T_{HS}}}\cdot \frac{\Delta S+\Delta S_{I}}{\Delta S}\cdot \frac{G_{T}\sqrt{T_{HS}}+\Delta S\left(\sqrt{T_{HS}}+\sqrt{T_{CS}}\right)}{G_{T}\sqrt{T_{CS}}-\left(\Delta S+\Delta S_{I}\right)\left(\sqrt{T_{HS}}+\sqrt{T_{CS}}\right)}.$$

If $\Delta S_{I}=d_{I}\Delta S$ (case a) of Section 2.3.1, the result is immediate by substitution in Equation (36). For the two other cases (b and c), the results will be numerical and considered hereafter in Section 3.3.

An interesting result coming out from Equation (36) is the *COP* at *endo-reversible condition*, namely, when $\Delta S_{I}=0$:

(38) $$\;\frac{1}{COP\;(\min\left|W_{endo-rev}\right|)}=\frac{T_{HS}}{T_{CS}}\cdot \underset{F’}{\underbrace{\sqrt{\frac{T_{CS}}{T_{HS}}}\cdot \frac{G_{T}\sqrt{T_{HS}}+\Delta S\left(\sqrt{T_{HS}}+\sqrt{T_{CS}}\right)}{G_{T}\sqrt{T_{CS}}-\Delta S\left(\sqrt{T_{HS}}+\sqrt{T_{CS}}\right)}}}-1.$$

It is important to note that the *correction factor F’ does not vanish in the endo-reversible case* when the coupling is considered.

It appears from Equation (38) that, for a given ΔS (existence condition of the cycle), the COP at minimum energy expense is strongly influenced by the value of ΔS in comparison with *G_T_*’s influence. The equilibrium thermodynamics limit is recovered only when ΔS/*G_T_* is small (or tends to the zero limit).

Equation (38) constitutes a new upper bound for refrigerator *COP* at minimum energy expense.

A second important consequence concerns the total entropy production of the system $\Delta S_{IS}$, whose value corresponds to the following:

(39) $$\Delta S_{IS}=\Delta S\left[\frac{\Delta S}{G_{c}+\Delta S}+d_{I}+\frac{\Delta S\left(1+d_{I}\right)}{G_{H}-\Delta S\left(1+d_{I}\right)}\right].$$

This function increases with *d_I_* and ΔS, for given *G_H_* and *G_C_* values, but it also presents an extremum when

(40) $$G_{C}^{*}=\frac{G_{T}-2\left(1+d_{I}\right)\Delta S}{2+d_{I}},$$

(41) $$G_{H}^{*}=\frac{G_{T}+2\Delta S}{2+d_{I}}.$$

The comparison of optimal expressions given by Equations (33), (34), (40), and (41) clearly shows that the optimal allocation of the heat transfer conductances differs when the energy expense minimization or minimum entropy production of the system is considered. Thus, in the first case, the dependence on the temperature appears in Equations (33) and (34), while it is missing in the second case from Equations (40) and (41). Consequently, *the theorem of entropy production minimization does not hold for refrigerator* (or, more precisely, for reverse cycle machine).

The optimum of entropy production is expressed as follows:

(42) $$Opt\;\Delta S_{IS}=\Delta S\left[d_{I}+\frac{\Delta S\;{\left(2+d_{I}\right)}^{2}}{G_{T}-d_{I}\Delta S}\right].$$

The corresponding value for endo-reversible case (*d_I_* = 0) is straight forward:

(43) $$Opt\;\Delta S_{IS_{endo-rev}}=\frac{4\Delta S^{2}}{G_{T}}.$$

The value is quadratically increasing with Δ*S* and decreasing with *G_T_*. Note that the equilibrium thermodynamic limit (reversibility) is retrieved when Δ*S*/*G_T_* tends to zero.

### 3.3. Modelling with Dependence on Temperature of the Production of Entropy

This dependence is defined by Equations (28) and (29).

#### 3.3.1. Production of Entropy Depending only on Temperatures

In this case introduced by Equation (28), *T_C_* remains a simple expression given by Equation (25). For *T_H_*, one gets the following:

(44) $$T_{H}=\frac{G_{H}-\Delta S+s_{IL}T_{C}-\sqrt{\Delta }}{2s_{IL}}\cdot T_{HS},$$

with $\Delta ={\left(G_{H}-\Delta S+s_{IL}T_{C}\right)}^{2}-4\;s_{IL}G_{H}T_{HS}$.

*T_H_* appears depending on *T_C_*, thus the optimization must be numerical.

Anyway, for low irreversible systems ($4\;k_{IL}G_{H}T_{HS}\ll {\left(G_{H}-\Delta S+s_{IL}T_{c}\right)}^{2}$), it is possible to derive an acceptable simple approximate solution expressed as follows:

(45) $$T_{H}≅\frac{G_{H}T_{HS}}{G_{H}-\Delta S+s_{IL}T_{C}}.$$

Considering the associated *W* value to this approximate solution confirms that the energy expenditure is increasing with Δ*S* and *s_IL_*, and decreasing with *T_C_*. The optimal distribution of *G_H_* and *G_C_* remains to be numerically sought (it is in due course).

#### 3.3.2. Production of Entropy Depending both on Intensity and Extensity

When $\Delta S_{I}$ corresponds to Equation (29), the expression of *T_C_* remains that given by Equation (25).

It is easy to transpose Equation (44) to obtain the new expression of *T_H_* associated to the new law of entropy production given in Equation (19):

(46) $$T_{H}=\frac{G_{H}-\Delta S\left(1-k_{IL}T_{C}\right)-\sqrt{\Delta }}{2k_{IL}\Delta S},$$

with $\Delta ={\left[G_{H}-\Delta S\left(1-k_{IL}T_{C}\right)\right]}^{2}-4\;k_{IL}G_{H}\Delta S\;T_{HS}$.

*T_H_* is always correlated to *T_C_*, but for low irreversible systems $4\;k_{IL}G_{H}\Delta S\;T_{HS}\ll {\left[G_{H}-\Delta S\left(1-k_{IL}T_{c}\right)\right]}^{2}$, it exists again as a reasonable approximation:

(47) $$T_{H}≅\frac{G_{H}T_{HS}}{G_{H}-\Delta S\left(1-k_{IL}T_{C}\right)}.$$

This approximated result corresponds to an energy expense always increasing with $\Delta S,\;k_{IL}$ and decreasing with *T_C_*.

The optimal energy conductance allocation remains a numerical one.

**Figure 5:**
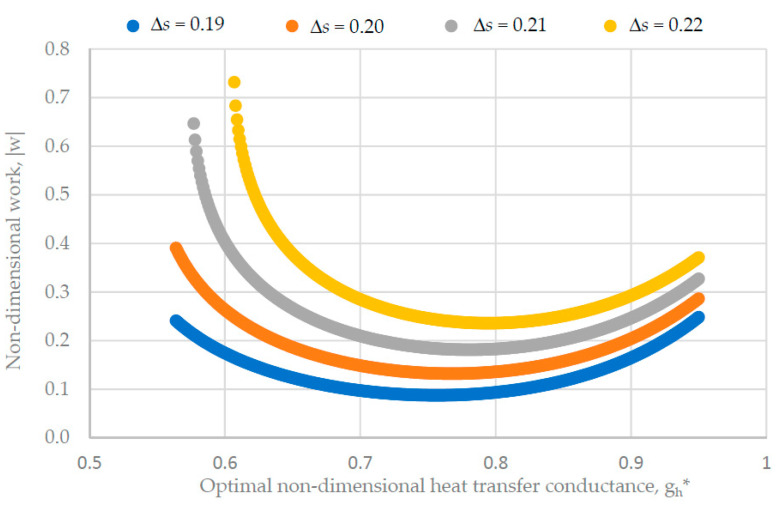
Non-dimensional work variation versus non-dimensional heat transfer conductance for different values of the non-dimensional reference heat transfer entropy and *k_IL_* = 0.001.

Some results of this numerical optimization are shown in Figure 5 for the case corresponding to entropy production dependence on both intensity and extensity.

Figure 5 illustrates the variation of the non-dimensional mechanical work expense with the optimum non-dimensional heat transfer conductance at the hot side, when the non-dimensional heat transfer entropy varies. The non-dimensional form of the involved property corresponds to the following:

(48) $$\left|w\right|=\frac{\left|W\right|}{G_{T}T_{0}},\;\;\;\;\;\;\;\;\;\;\;\;g_{h}^{*}=\frac{G_{H}^{*}}{G_{T}},\;\;\;\;\;\;\;\;\;\;\;\;\Delta s=\frac{\Delta S}{G_{T}}.$$

The curves on the figure clearly show the existence of a minimum energy expense |w| for each value of the optimum non-dimensional heat transfer entropy Δ*s*, and this minimum slightly slides to higher values of $g_{h}^{*}$ (from 0.75 to 0.8) as Δ*s* increases (from 0.19 to 0.22). Moreover, as Δ*s* increases, the variation range of left side values of $g_{h}^{*}$ decreases (from 0.56–0.95 to 0.61–0.95), together with a sharp increase in energy expense |w|. Thus, it is obvious that an operation regime with small values of $g_{h}^{*}$ should be avoided even for small values of Δ*s*.

Figure 5 also shows the strong influence of the reference heat transfer entropy on the minimum energy expense because, for $g_{h}^{*}$ values near this minimum |w|, its non-dimensional value increases 2.5 times (from 0.1 to 0.25) for a Δ*s* increase of 0.158 (from 0.19 to 0.22).

A detailed study is in due course near to the authors.

## 4. Conclusions and Perspectives

This paper has reported of gradual generic models of refrigerator, starting from the reverse Carnot cycle followed by Chambadal’s machine configuration and, finally, the Curzon-Ahlborn refrigerating machine.

The main original contributions of the paper are as follows:

- The comparison with these various models taking account or not the supplementary constraint due to coupling of the machine (cycle) with the cold source and hot sink. This introduces new results depending not only on intensities (temperatures), but also on extensities (reference heat transfer entropy Δ*S*, and production of entropy Δ*S_I_*).
- The comparison of the results obtained for three kinds of entropy production laws for the machine. The mechanical energy expense is always an increasing quantity of the irreversibility parameters (*d_I_*, *s_I_*, *k_I_*), but also of Δ*S*, the reference heat transfer entropy.
- An optimal allocation of heat transfer conductances, *G_H_* and *G_C_*, was confirmed and completed in the case of Curzon-Ahlborn configuration, whichever form of the entropy production law is considered. Analytical solutions were obtained when Δ*S_I_* is a constant parameter or proportional to Δ*S*, the reference heat transfer entropy (extensive proportional entropy production model). Complementary studies for other entropy production laws are in due course depending on available intensity, or extensity, or both. In any case, the optimal allocation of *G_H_* and *G_C_* differs if the minimum of energy expense, or the minimum of the system entropy production, is considered.
- The *COP* associated with the minimum of energy expense was obtained and its corresponding expression for the endo-reversible configurations was determined. It constitutes a new expression of the upper bound for the refrigerator performance (Equation (36)).
